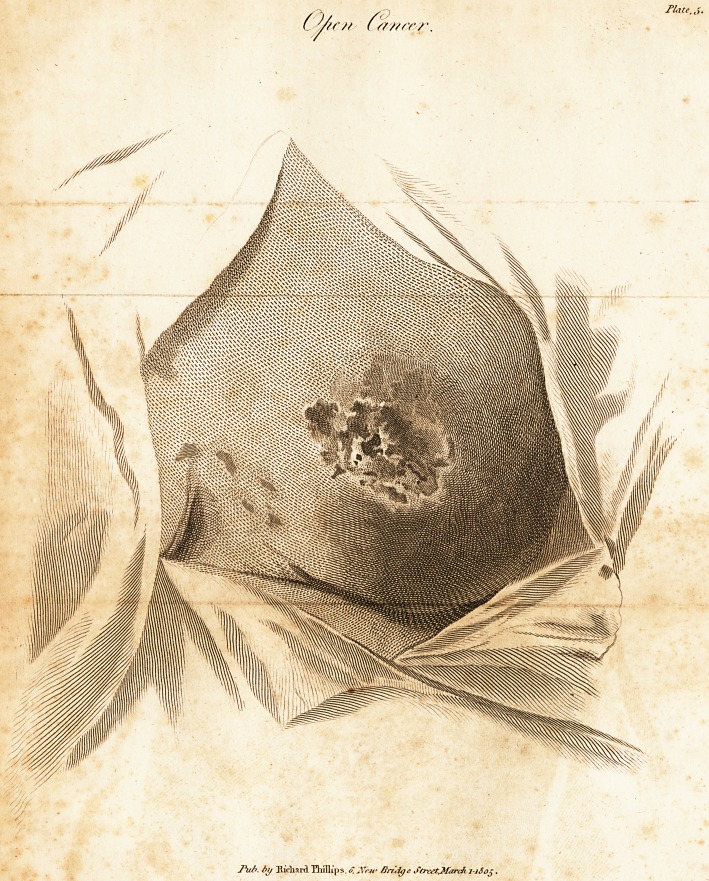# Observations on Select Subjects in Surgery

**Published:** 1805-04-01

**Authors:** 


### Medical and Phyſical Journal.

VOL. XIII.] APRIL 1, 1805. [NO. LXXIV.

*Printed for R. PHILLIPS, by W. Thorne, Red Lion Court, Fleet Street, London.*

### OBSERVATIONS ON SELECT SUBJECTS IN SURGERY.

BY MR. SIMMONS.

( Continued from pp. 97—103. )

### ON STEATOMA.

AS the end of all arrangement is utility, the practical advantage of classifying tumours, whose treatment is similar, is not very obvious. Conversely, it is much more valuable to know that certain disorders, though diversified in their external character, are in their nature so analagous, as to yield to a like mode of treatment. Of this sort is the steatoma, a disease akin to several others, which, in their advanced stage, can only be effectually removed by the knife.

This species of tumour originates from the extravasation of fluids into the cellular membrane, which, instead of being absorbed, or converted into pus, receive an imperfect organization by very slow degrees. The growth of the steatoma, though slow, is progressive, until, in some cases, it has arrived to a large size, much larger than what is here recorded. From its low sensibility, it is not apt to inflame, and is rather inconvenient from its bulk than painful. It is confined to no particular situation, though it has happened to me to meet with it most frequently on the back and thighs.

In the early stage of the steatoma, it may be worth while to try the effect of topical remedies, such as stimulants by friction and pressure, in the hope to destroy its feeble vital powers, and render it fit for absorption. Where these and electricity fail, excision is fully warranted.

In the extirpation of a steatoma of a moderate size, a longitudinal incision will commonly give sufficient room to the operator; but, when it is of a large magnitude, it will be better to rescind a portion of the integument, or it will pucker by its super-abundance, and hinder an early union. Agreeably to this opinion, I acted in the following case.

A gentleman, near sixty years of age, had a large steatoma, which had sprung from a blow, and increased slowly for thirty years. It was situated upon his neck and shoulders, and occupied a space from the occiput down to the lower angles of the scapulæ, and, anteriorly, was descending towards the clavicle on each side. Alarmed by this, he determined upon its removal, although it was neither inflamed nor painful, and only troublesome to him by its unwieldy size. I began the operation by making two corresponding incisions the whole length of the tumour, so as to include an oval portion of the skin, which was left adhering to it, and then I dissected out the whole, which took up about ten minutes. The arteries divided in the operation, were not secured until now, for I wished him to lose as much blood as was likely to hinder much subsequent inflammation; and he lost, I believe, about twenty ounces. The hæmorrhage however would have been more considerable, but for the activity of my assistants. Before the operation, I had conceived the idea of at once obliterating this immense cavity, by an immediate union between the upper and under surfaces, whence the tumour had been separated; accordingly, the lips of the wound were held together by about thirty stitches; long broad stripes of adhesive plaster were applied in every direction; and over these, bolsters of tow were supported by a moderately tight bandage.

At the first dressing, which took place on the third day, I had every reason to believe that union by the first intention was throughout complete; and in a week he was able to leave the house.

The tumour weighed six pounds two ounces avoirdupois; and the engraving will shew, had the true lobulated appearance of the steatoma.

The only thing remarkable, to which I hope for the attention of the reader in this case, is the method of dressing that was here adopted with full success, and which, upon further experience in cases nearly similar, I can recommend to the adoption of others.

Many years ago, I removed a steatoma that hung pendulous, near a foot in length, from the left labium pudendi, in a young unmarried lady. She was insane at the time, and it is not improbable but her delicacy concerning it, it, had proved the cause of that awful visitation; at least, soon after its extirpation, her senses were restored to her; and continued perfect when I last heard of her, at the end of several years. The wound united by the first intention in this case also.

### Sarco-Epiplocele.

A middle-aged man, of sober and industrious habits, requested me to look at a swelling on the left side of his scrotum. On examination, it appeared to be an enlargement of the testicle, together with an induration in the epididymis, and an unusual fullness in the cord. The account which he gave of himself, was, that a few months before, he first perceived a small swelling about as big as a walnut, and being told that it was a rupture, himself and others had made strong and frequent ineffectual attempts to reduce it; and these failing, a truss, the pad of which consisted of a solid piece of wood, was placed over the swelling to waste it away, or at least to prevent any further descent of it into the scrotum. At this time, the testicle itself was as large as a middle-sized orange, equal on its surface, and free from pain, even after moderate handling. Hopes of its dispersion being entertained, he was advised to try discutient remedies; and in the use of these, he persisted for some time, but without any advantage. Indeed, the swelling of the testicle augmented very rapidly a short time previously to the operation, which, therefore, at his own request, I performed upon him on the 30th of March, 1804. In cutting down to secure the spermatic cord, a portion of omentum that lay anterior to it, immediately protruded; on turning up the omentum, the cord appeared quite sound a little after its exit from the testicle; here, therefore, the ligature was applied round the whole cord at once, which was then divided just below. After this, he felt very little pain in the excision of the testicle. The state of the omentum was next enquired into; when the supposed indurated epididymis, which indeed was entirely wanting, turned out to be a diseased portion of omentum, a large quantity of which had insinuated itself into the tunica vaginalis, but did not adhere to the testis. As none of it could be returned, it was removed close to the ring with a pair of scissars; however, without the ensuing of any hemorrhage, so that no ligature was required upon any of its vessels. The lips of the wound were held in contact by the interrupted suture; and the customary dressings applied.

The morning after the operation, the pulse being hard and full, he lost sixteen ounces of blood from the arm; and as it was sizy, and the pulse would bear it, the bleeding was repeated in the evening to the quantity of twelve ounces. On the following morning, his bowels were copiously emptied by a saline purgative. On the third day, the tension and redness in the course of the cord were pretty considerable; and as his pulse did not indicate general bleeding, fifteen leeches were laid upon the part; and, early the next morning, the purgative was repeated. On the fifth day, suppuration had commenced; and he began with the bark. And, on the eleventh, the ligature came away; after which the discharge gradually subsided, and, in a month, the wound was completely healed.

This case proved more complicated in the treatment of it, than was at first imagined. On the supposition of an intestinal hernia, he was enjoined to keep his bed for several days prior to the performance of the operation, and, by his own account, there was reason to believe that something that had prolapsed into the scrotum, had reascended into the abdomen, during his confinement. But this could not have been the omentum; because, after it had become indurated, it could not have quitted the tunica vaginalis. It was most likely therefore a turn of intestine, that had slipped down occasionally behind the omentum.

The morbid alteration which the testicle itself had undergone, was evidently of a scrofulous nature; hence some may be disposed to carp at the employment of the term *sarcocele*, as being contrary to its usual technical acceptation. But, I shall not dispute about a name. The inner structure of the testicle was evidently *carnous*; yet, so elastic on pressure, as, a few days before the operation, to mislead us to the introduction of the trocar, conjecturing that there might be water, in order to ascertain the real state of the testicle; however, no water was evacuated, nor was he at all prejudiced by the attempt.

He first perceived the descent of this testicle in his thirty-seventh year; and looking upon it as a defect, through mistaken and false delicacy, by endeavouring to conceal it, he unfortunately fell into improper hands. Much of the mischief that ensued may doubtless be ascribed to the rude violence with which it was treated; at least, the patient himself always declared, even while I was trying to preserve it, that it had been so ill-used that nothing could save

Scrofulous Testis.

Fig. 1.

Diseased portion of Omentum.

Fig. 2.

Printed for Richard Phillips, New Bridge St. 1805

*Pulmonary Ossifications.*

*Scirrhus Testicle.*

*Printed for* Richard Phillips, *6, New Bridge St. 1805.*

save it; and, consistently with this declaration, he solicited its removal from the first.

Such instances as this are, I apprehend, not very common, though the works of practical writers are not without records of the injury sustained by mistaking a testicle, while detained in the groin, for a hernia or bubo. Certainly, in the present case, the descent was unusually late; and it might be amusing, if not profitable, to enquire a little into the cause of so singular a phenomenon; for, as far I can recollect, no physiologist has yet investigated the subject with much precision. We have reason to believe, that the peculiar mode of existence, and functions assigned to every organ, have an intimate relation to its internal structure; and that if by any accident the organization of a part should be materially altered, a reciprocal alteration of function will necessarily ensue. Agreeably to this position, the detention of the testicle within the abdomen, for so many years beyond the usual time of the descent of it into the scrotum, may be explained. I have before observed, that the epididymis was wanting; nor is that all, for the vas deferens, which should have sprung from the upper extremity, was inserted into the very bottom of the testis, and from this point took a direction round the body of the gland to the upper extremity, and from thence passed forward through the ring with the rest of the cord. To this *lusus naturæ* I should be disposed to ascribe the irregularity of the descent in this case; not, however, that the deviation was such as to hinder secretion, or obstruct the office of the excretory duct.

To represent the circumstances just mentioned, as well as the appearances assumed by scrofula in the testicle, when laid open by a longitudinal incision, I have caused a coloured drawing to be made. And also a second, to depict the indurated portion of omentum: in both, the suffused redness was probably owing to the trocar, which must have passed through the omentum into the body of the testicle.

### Scirrhus Testicle.

To elucidate the history, or convey in words a distinct idea of the alteration of structure produced in a glandular part by scirrhus and cancer, may justly be reckoned a vain attempt; for, as far as language is descriptive of morbid appearances, the subject, in my opinion, is already exhausted. Perhaps something may be done by the art of painting, but even of this my expectations are but moderate. Some time anterior to the above case of scrofulous testis, I had come to the resolution to procure a series of coloured drawings relative to scirrhus and cancer, and I have introduced that here, as introductory to the two following, which are all I at present possess. In the first is shewn the morbid appearance exhibited by scirrhus in a testicle, which was extirpated from a middle-aged man, soon after the former operation. This disease had existed for seven years, yet, until a few months before the excision was performed, the testicle was but inconsiderably enlarged, though to the touch it presented a stony hardness, was craggy, and painful after handling. Subsequently the pain increased, with exacerbations at irregular intervals, and the enlargement proceeded very rapidly. Still, as the cord was apparently free from disease, I proposed the operation to him without delay, to which he readily submitted; and the wound was healed within a reasonable time. But when the wound was nearly closed, a severe pain fixed in the right hip, and a tensive opake swelling pervaded the thigh and leg on the same side; these however yielded to leeching, a blister over the pained part, and fomentations and spirituous embrocations generally to the extremity; and, lastly, as the tensiveness abated, a moderately tight roller was duly applied.

### Open Cancer.

The second drawing exhibits a specimen of open cancer, which occurred in an elderly woman who had borne many children The disease was in the state represented when she first offered at the Infirmary; the axillary glands were much enlarged, and the whole arm was tumefied. In a former instance of ulcerated cancer of the mamma, the tumefaction began in the hand, and ascended by degrees up the arm to the shoulder; and the cool or tensive state of the skin of the hand over the knuckles, very certainly indicated the calm, or irritated state of the breast itself. In the present case, however, the direction of the swelling was reversed, and it descended from the shoulder down to the hand, without any distinguishing affection of the skin over the knuckles

[To be continued.]

### Open Cancer.

Pub. by Richard Phillips, 6, New Bridge Street, March 1. 1805.

## Figures and Tables

**Figure f1:**
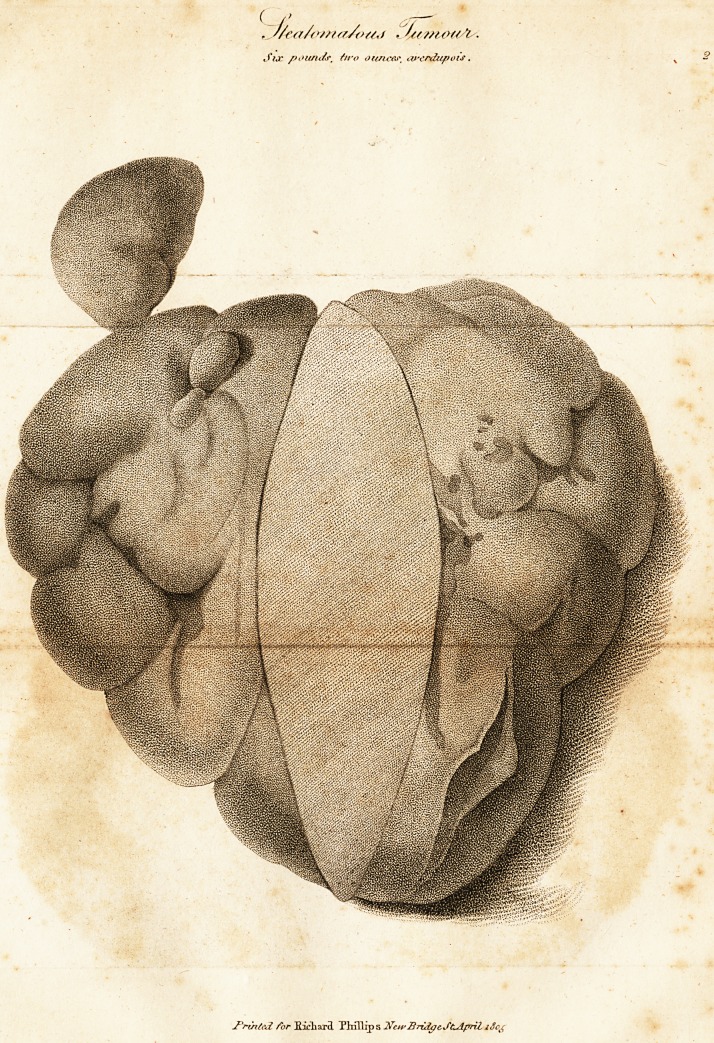


**Fig.1. Fig.2. f2:**
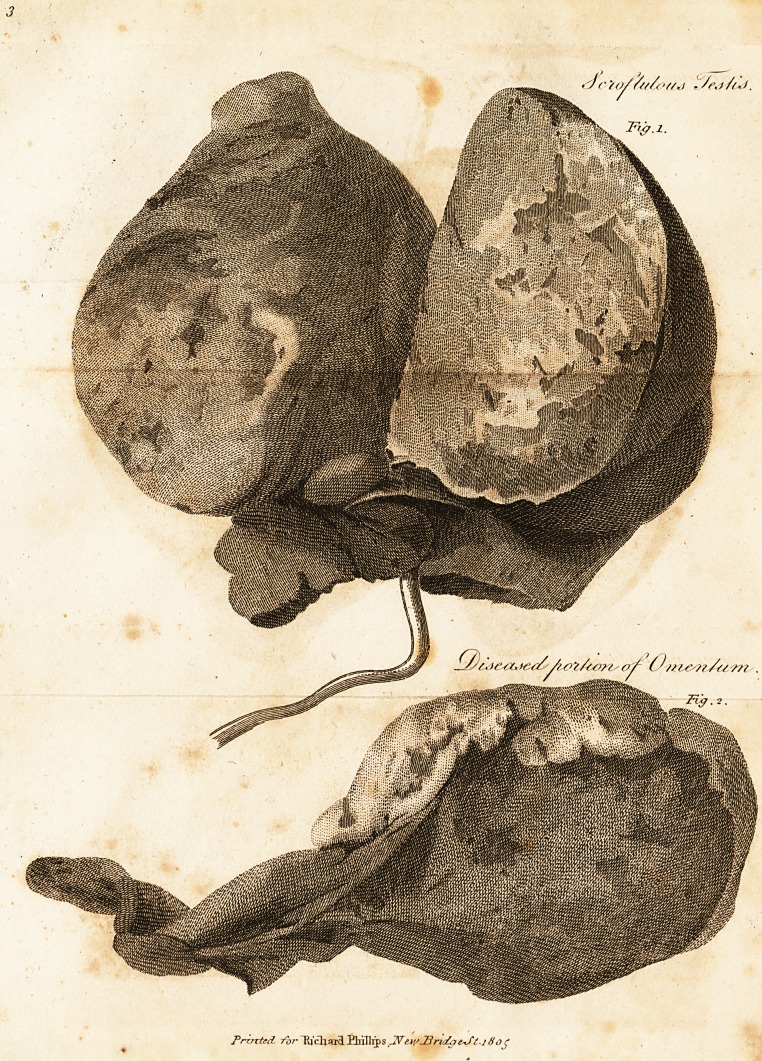


**Figure f3:**
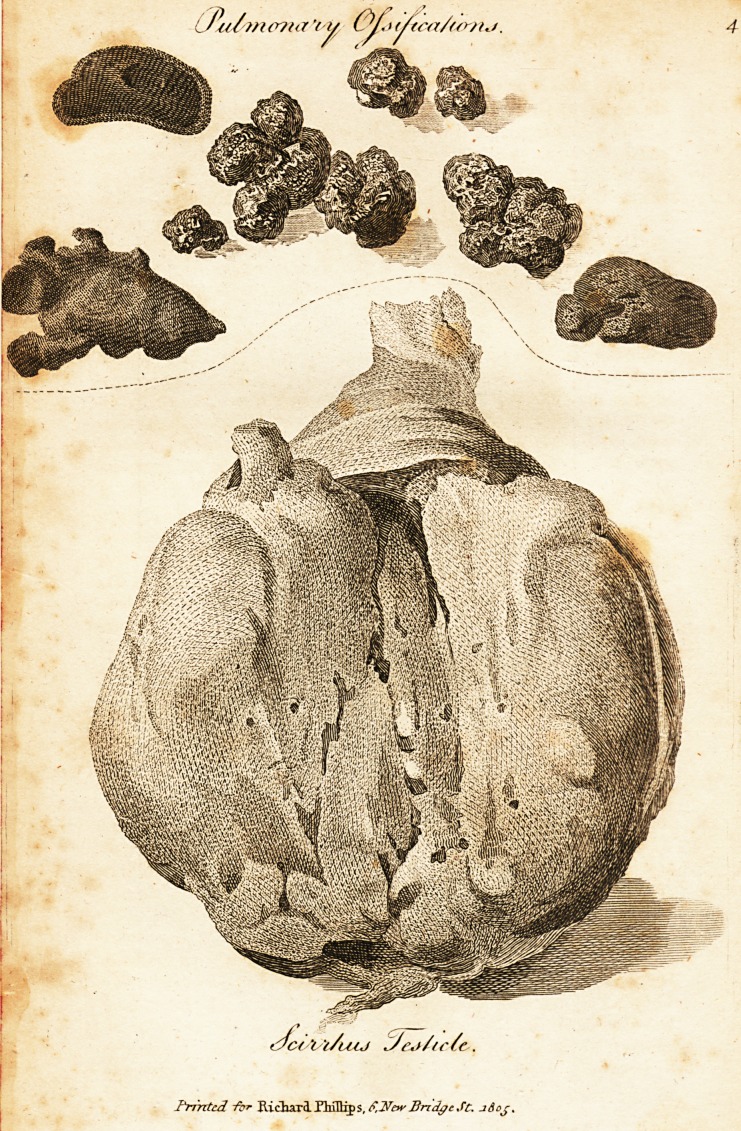


**Figure f4:**